# Pandemic Pause: Systematic Review of Cost Variables for Ambulatory Care Organizations Participating in Accountable Care Organizations

**DOI:** 10.3390/healthcare9020198

**Published:** 2021-02-12

**Authors:** Cristian Lieneck, Eric Weaver, Thomas Maryon

**Affiliations:** 1 School of Health Administration, Texas State University, San Marcos, TX 78666, USA; 2 Accountable Care Learning Collaborative, Western Governors University, Salt Lake City, UT 84107, USA; eric.weaver@wgu.edu; 3 Healthcare Policy, Economics, and Management School of Community and Rural Health, The University of Texas Health Science Center at Tyler, Tyler, TX 75708, USA; thomas.maryon@uthct.edu

**Keywords:** accountable care organizations, value-based care, ambulatory care, outpatient care, medical clinic, cost

## Abstract

Ambulatory health care provider organizations participating in Accountable Care Organizations (ACOs) organizations assume costs beyond typical practice operations that are directly associated with value-based care initiatives. Identifying these variables that influence such costs are essential to an organization’s financial viability. To enable the U.S. healthcare system to respond to the COVID-19 pandemic CMS issued blanket waivers that permit enhanced flexibility, extension, and other emergency declaration changes to ACO reporting requirements through the unforeseen future. This relaxation and even pausing of reporting requirements encouraged the researchers to conduct a systematic review and identify variables that have influenced costs incurred by ambulatory care organizations participating in ACOs prior to the emergency declaration. The research findings identified ACO-ambulatory care variables (enhanced patient care management, health information technology improvements, and organizational ownership/reimbursement models) that helped to reduce costs to the ambulatory care organization. Additional variables (social determinants of health/environmental conditions, lack of integration/standardization, and misalignment of financial incentives) were also identified in the literature as having influenced costs for ambulatory care organizations while participating in an ACO initiative with CMS. Findings can assist ambulatory care organizations to focus on new and optimized strategies as they begin to prepare for the post-pandemic resumption of ACO quality reporting requirements once the emergency declaration is eventually lifted.

## 1. Introduction

### 1.1. Rational

The novel Coronavirus (COVID-19) global pandemic has impacted the United States healthcare industry dramatically. Since March 2020, treatment protocols and access to care have been affected, challenging the quality of care provided to patients of all acuity levels [1,2,3]. This deferment of care exacerbated chronic illnesses and related symptoms, and a reduction of routine and preventative care continues to hide as a secondary concern behind the ongoing COVID-19 virus surges and initiatives to increase physical distancing and other public health measures [4,5]. To assist health care organizations in treating patients requiring immediate, acute care related to the pandemic, the Centers for Medicare and Medicaid Services (CMS) has relaxed the Quality Payment Program (QPP) reporting requirements for Accountable Care Organizations (ACOs) [6]. Further, recent provider practice pattern shifts from the pre-COVID-19 values-based quality metric reporting system for ACO-involved organizations offers an assessment opportunity to identify key cost-influencing variables within the ACO environment.

### 1.2. Objectives

Since the Patient Protection and Affordable Care Act (2010) and the updated value-based payment reform initiatives of the Medicare Access and CHIP Reauthorization Act (2015), many ambulatory care organizations have participated in ACOs. These organizations have assumed risk-based reimbursement challenges with varying results. The ambulatory care management team, confronted with reducing the cost of care while maintaining and improving patient quality of care and treatment outcomes, experience additional operational costs to their organization when participating in ACOs. The current QPP reporting accommodations for these organizations allows for a reflection on costs associated with ambulatory care (outpatient) organizations participating in ACOs for the period between 2015 and early 2020 significantly affected by MACRA (2015). The identification of variables during the evaluation period that both increase and reduce costs associated with ambulatory care organizations while currently participating in ACO reimbursement models since the last QPP updates can assist outpatient organizations in their resumption of routine operations and best-practice process design once the pandemic’s effect on the industry has subsided.

## 2. Methods

This systematic review was guided by the Preferred Reporting Items for Systematic Reviews and Meta-Analysis (PRISMA). Literature for this review related to accountable care organizations and ambulatory care provider organizations was obtained from four separate databases: (CINAHL) Complete, Academic Search Ultimate, Business Source Ultimate via the Ebson B. Stephens Company (EBSCO host) and PubMed (which queries MEDLINE). Overall, four databases were utilized to broaden the search due to an initial observation of limited publications meeting the search criteria. Peer-reviewed publications specific to ambulatory care organizational costs directly related to an ACO model’s reimbursement is limited and therefore important to be identified as post-pandemic preparations for the return to routine operations begin.

**Figure 1:**
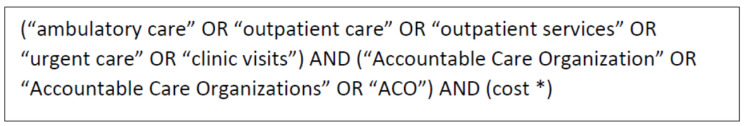
Research database search string as populated by the use of MeSH exploding vocabulary and Boolean search operators.

The researchers focused on ambulatory care/outpatient organizations and an investigation into costs associated with accountable care organization participation. Medical Subject Headings (MeSH) is the National Library of Medicine controlled vocabulary thesaurus utilized to index research articles for PubMed (MEDLINE) and was used to identify key words in the search. Boolean search operators were used to ensure proper word/phrases to capture all applicable literature for the sample as indicated by MeSH terms and follow-on “exploding” terminology specific to ambulatory care organizations. The variable of “cost” was truncated in the search string to assist in potential variations of the term (Figure 1).

### 2.1. Inclusion Process

To be included in the sample, publications must have occurred between 1 January 2015 and 1 October 2020. This specific publication date range criteria were utilized to ensure studies identified were appliable to the most-recent Medicare Access and CHIP Reauthorization Act (2015) value-based reporting criteria as implemented by the CMS Alternate Payment Method (APM) Quality Payment Program (QPP) for ACOs [7]. Editorials, government reports, letters to the editor, or other studies not based upon empirical evidence were not considered in this review. Full text was not included in the initial search criteria in an attempt to simply locate the applicable literature (citations) and permit the researchers to conduct follow-up on individual article searches for full text of identified citations via their three separate research institutions’ online library databases (therefore increasing review validity and also sample size). The researchers were able to locate 100% of the final sample in full-text format for follow-on extensive review.

Studies in this review had to involve ambulatory care (outpatient) organizations and measurable costs as associated with their participation in a Medicare ACO alternative payment model. Specific facilitators and barriers to cost inflation and/or reduction were intended themes to be identified, as previous reviews of literature in this specific industry segment and related costs associated with accountable care organizations remains limited. Many publications included in the review also address costs incurred by network hospitals and other integrated systems in addition to ambulatory care service locations. While the ACO program and its related value-based and shared-savings criteria have been adjusted over time, this review focused specifically on cost evidence as applied to ambulatory care organizations in order to review their investments in value-based care, as compared to solely hospitals and other large hospital networks/ancillary systems.

This study’s information came from secondary data sources (library research database). All of the literature included in this research is publicly available and any individual research subjects (if present) are unidentifiable. As a result, this systematic review qualifies under “exempt” status in 45 Code of Federal Regulations (CFR) 46. An institutional review board review was not required, and no consent was necessary.

### 2.2. Exclusion Process

**Figure 2:**
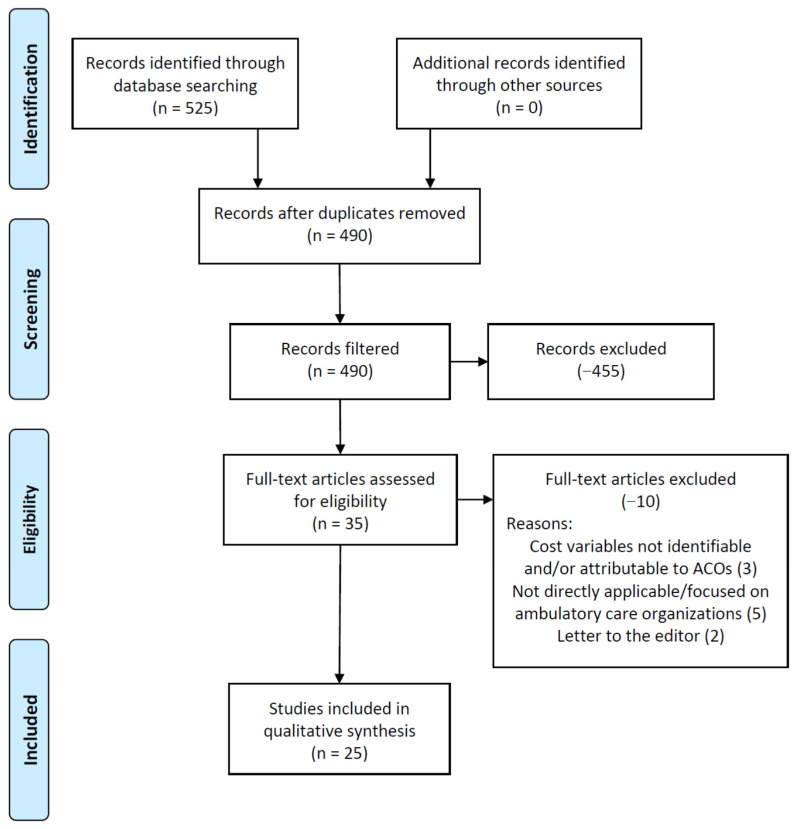
Preferred reporting items for systematic reviews and meta-analysis (PRISMA) figure that demonstrates the study selection process.

Figure 2 demonstrates the article exclusion process, beginning with the initial research database searches and ending with the final literature sample (n = 25). Initially, 525 articles met the primary search criteria, addressing the variables of both ambulatory care, accountable care organizations, and cost. While four research databases were utilized in this study to help increase the literature sample size, a consequence of this decision was a high frequency of duplicate articles identified (35 total duplicates). Upon filtering these results for the study’s specified date range to further identify costs as applicable to the MACRA (2015) policy implementation period (pre-pandemic), and also English-only, academic/peer-reviewed journals, 455 articles were excluded from the study, leaving 35 remaining in the study sample. These remaining 35 articles were then downloaded by the research team (full text) and thoroughly reviewed. All researchers reviewed the literature to ensure each was germane to the study’s initiative.

Upon completion of the final screening process, the research team decided to remove an additional 10 articles from the sample for the following reasons: letters to the editor (−2 articles), cost variables not identifiable and/or attributable to ACOs (−3), and not directly applicable/focused on ambulatory care organizations (−5). These measures were conducted by team meetings (via webinar) by the research team on multiple occasions and all researchers reached a consensus (100% agreement) on article exclusions and the solidification of the final literature sample (n = 25).

The researchers have significant experience managing in the ambulatory care environment and the decision was made to review all the literature sample independently. Further, individually identified underlying themes (constructs) as related to cost influencers and costs preventions as experienced by ambulatory care organizations participating in ACOs were identified. To further categorize and synthesize results and to increase inclusion of findings, researchers further classified identified themes into final study variables related to costs associated with ambulatory care organizations participating in ACOs (secondary/final theme) in complete agreement. Finally, quality assessment of the literature in the sample was conducted using the Johns Hopkins Nursing Evidence-Based Practice Model (JHNEBP) to further identify types of studies identified.

## 3. Results

According to the JHNEBP classification model, 76% (19) of the identified articles were categorized as Level 2, or quasi-experimental studies. These studies often used a pre- and post-assessment or group difference analysis of an ACO’s patient outcomes and related organizational cost information. Other identified articles fell within Level 3 studies classified as non-experimental in nature, yet still met the criteria for this study and assessment of costs incurred or prevented by ambulatory care organizations participating in ACOs. JHNEBP Levels 4 and 5 (studies related to expert opinion and/or panel recommendations) were not included in the study (Table 1).

Additionally, Table 1 also addresses facilitators related to cost prevention strategies for ambulatory care organizations participating in ACOs. Cost reduction findings and/or experiences by participating outpatient care organizations are summarized, while cost reduction barriers, either an inability to decrease cost or possibly even contribution towards increasing costs are notated. While it may be assumed that an inability of an outpatient care organization to correctly implement the study’s identified cost reduction facilitators would lead to increase cost, additional identified barriers to cost prevention/reduction (leading to increases in costs) are also presented in Table 1.

**Figure 3:**
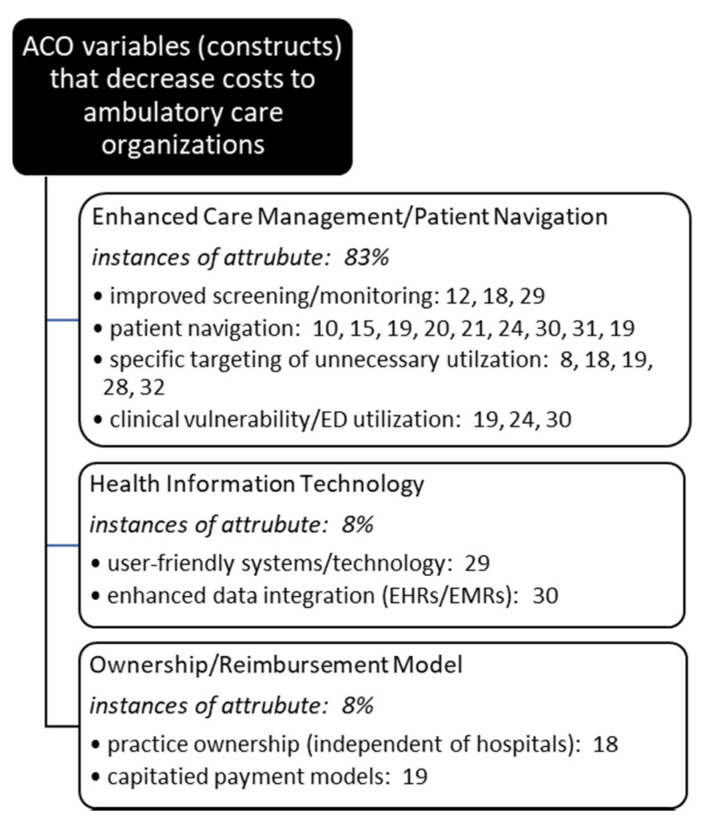
Occurrences of underlying themes (constructs) identified in the literature decreasing costs in ambulatory care organizations participating in ACOs.

**Figure 4:**
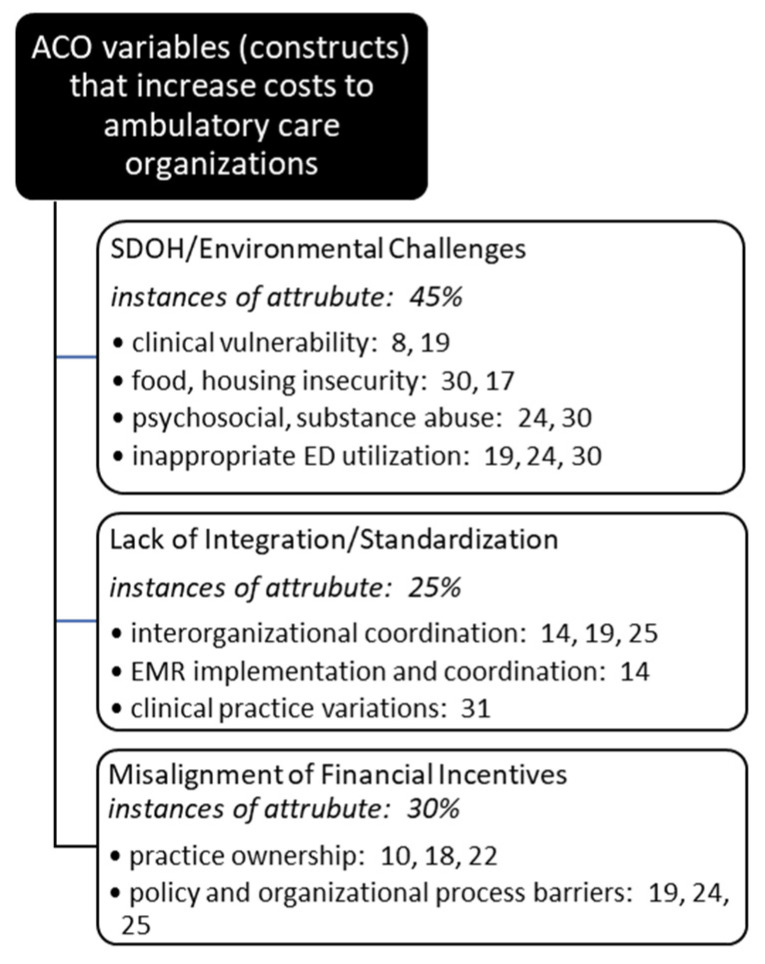
Occurrences of underlying themes (constructs) identified in the literature increasing costs in ambulatory care organizations participating in ACOs.

Once all articles were reviewed and underlying themes (constructs) identified and finalized, researchers met to review individual results and collectively decide upon a single affinity matrix. Agreement was reached on cost influencing and cost prevention variables and article assignments, or coding. Each had to agree on each identified article’s inclusion into each thematic category. Article inclusion into thematic categories was not mutually exclusive, and often a single article met criteria for multiple cost influencer and/or reducer categories (Figure 3 and Figure 4).

## 4. Discussion

The systematic review identified key the variables that influence cost. Understanding and addressing these variables can play an important role for the ACO to contribute to organizational financial viability. These variables identify interventions that can help reduce costs when implemented as well as inform ACOs on key areas that need to be addressed and if left unmanaged can lead to increased costs to the ACO.

### 4.1. Cost Reduction: Enhanced Care Management/Patient Navigation

Most consistent with CMS initiatives and the ACO model patient-centered care focus, the incorporation of a patient care management program and patient navigation processes were identified as most prevalent in the literature to assist with cost reduction. This finding suggests that those outpatient organizations investing in patient-level quality of care/care coordination activities experience cost reduction along with improved clinical outcomes.

Care intensive, patient acuity-based programs were implemented by many of the outpatient organizations to support this initiative [11,13,24]. Such programs developed by these organizations specifically worked to identify indicators of potential current and/or future illness and cost drivers as related to specific diagnoses associated with high risk and therefore high-cost treatment options. Most noticeable and cost effective was an outpatient program’s ability to custom tailor these initiatives to the specific communities they served and inherent characteristics specific to the various environment of care [24].

Investment in gathering community demographics and other characteristics is also cited as a best practice [14]. Such investment in market analysis, the health status of the population served, and making investments in addressing disparity leading gaps to improve health not only demonstrates an investment in population health but will also benefit the organization’s quality reporting efforts.

Patient education and the implementation and expansion upon patient self-care responsibilities contributed to significant cost reductions for outpatient organizations involved with the ACO model. Specifically, a program developed to identify those patients with the ability to provide their own self-care at home was initiated to support overall value-based care [12]. This initiative was also commonly identified in the literature as a contributing variable for outpatient ACO cost reductions with advanced telehealth treatment expansion initiatives [17,28].

The use of patient care navigators further contributed to identified cost reductions in the outpatient clinic literature. Such positions have been developed to assist patients (often virtually) during the global pandemic and beyond for both clinical and non-clinical initiatives [15,25]. Similar clinic-associated tasks conducted by employees and/or providers were identified as participating in such actions but not specifically identified as patient navigators [16]. Demonstrated cost reductions also involve the prevention of frequently experienced evaluation & management (E & M) visits for nursing home organizations [32].

### 4.2. Cost Reduction: Health Information Technology

The shift of clinical practice away from traditional silos of care to greater team focused care to achieve improved patient quality and cost benefits continues to be a healthcare delivery focus. User friendly Health Information Technology (HIT) is a critical enabler of this shift. HIT use across the clinical management continuum provides improved patient identification, clinical intervention tracking, outcomes monitoring tools, and the development of robust data sets to contribute to quality and process improvement efforts and ongoing identification of clinical best practices.

Aledade, a national network of independent practices has utilized a HIT platform to maximize the implementation and effectiveness of Medicare’s Annual Wellness Visits (AWV). This ACO has implemented user friendly HIT enablement to ensure consistent identification of high-risk beneficiaries, facilitation of visit scheduling, optimizing visit workflow, and to provide data tools for performance monitor and best practice identification [29]. Other ACOs are seeing the promise of technology use and data integration and sharing by showing improved outcomes in high-risk Medicaid populations by utilizing clinical, claims, health plan enrollment and demographic data, as well as social service data provided by community health workers. Improved outcomes for this ACO include reduction in Emergency Department (ED) visits and an increase in utilization of primary care [30].

### 4.3. Cost Reduction: Ownership/Reimbursement Model

A cost reduction initiative identified in the literature surrounded the type of ambulatory care organization (specifically to ownership), as well as potential affiliation and/or other external organizations that may participate in the respective ACO network. Several studies support the identification of physician-owned organizations as the most successful models in limiting costs associated with ACO participation [18,22]. Such observation also possesses an inherent alignment of financial incentives for associated ACO stakeholders.

Physician-led ACOs have been the most successful type of risk bearing entity in the health value landscape as demonstrated by public reporting of the Medicare Shared Savings Program (MSSP). MSSP performance year results have consistently shown that smaller, physician-led ACOs are more likely to earn shared savings than hospital-led or integrated hospital and physician group-led ACOs [33]. Physician-led ACOs have done better at achieving savings despite less access to capital, less experience managing risk, and less sophisticated HIT systems [33]. Keeping with past trends, ACOs led by physician groups saw higher levels of savings in the MSSP than those led by hospital systems, saving an average of $114 per beneficiary compared to just $61 for hospital led ACOs in Performance Year 2019 [34]. Physician-led ACO success is also seen in the Medicaid program which lends verifiability across different population types in promoting value-based care [17,20,26,30].

### 4.4. Cost Influencer: Social Determinants of Health/Environmental

It is commonly accepted that health and illness follow a social gradient and when that gradient is tilted towards a lower socioeconomic status there is a greater negative impact on health and higher cost. The complex issues that need mending in our social fabric will not be easily solved. ACOs actively experience the negative cost impact of social determinants on member health and need interventions to address until larger, more systemic issues are resolved.

In addition to medically complex conditions, mental health and substance abuse issues, and psychosocial issues make a significant contribution to patient cost and complexity. Social determinants such as food insecurity, homelessness, social disruption, or lack of social support play a key role in health and illness. These characteristics contribute to patients being identified as both high-risk and high need [24]. Given the high rate of emergency department and inpatient care among homeless populations, this consideration should be given priority by the ACO team with living arrangement/home situation status being both assessed and addressed [8]. Given the cost impacts of socioeconomic variables improving the documentation of socioeconomic indicators through an ACOs HIT platform to measure and monitor health disparities can facilitate proactive intervention and care coordination. [30].

### 4.5. Cost Influencer: Integration/Standardization Challenges

A lack of standardization of health care services provided by clinicians significantly contributed to increased costs experienced by ACO outpatient organizations [14,29]. Those organizations that focused on clinical pathways that were supported by industry best practices and other identified clinic protocols often utilized the electronic medical record and clinical decision support systems (CDSS) to help standardize practice patterns. Further, the lack of evaluating and incorporating industry system challenges (referral processes, follow-up post-acute care, and other between-provider organization collaboration) was cited as a significant cost influencer for the outpatient clinic [12].

### 4.6. Cost Influencer: Misalignment of Financial Incentives

A major hurdle for physician-led ACOs to effectively execute on a value-based operational agenda that will effectively lower overall costs for a managed population is the misalignment of financial incentives. This is especially true when participant practices are still operating in a predominantly fee-for-service business model that directly competes with the value-based care focus of the ACO. This misalignment of financial incentives between value and volume is difficult to reconcile without a clear understanding of any specific medical practice’s portfolio regarding payer mix with regard to risk-based reimbursement methods, or if/when the MSSP ACO contract transitions from pay-for-reporting to pay-for-performance measures—thereby necessitating investment in clinical transformation activities [10]. Given that the ACO program in Medicare appears to embed the assumption that large multispecialty organizations centered on hospitals will be best positioned to implement improved care delivery systems to lower costs [18], there could also be a misalignment in financial incentives due to practice ownership type. Research analysis of MSSP ACOs refutes the assumption that hospital-integrated practices are predisposed to advantage; however, as hospital-integrated ACOs did not produce savings in proportion to physician groups [22].

## 5. Study Limitations

As with any study, limitations exist. There is a lack of peer-reviewed research surrounding ambulatory care/outpatient organizations and their participation in ACOs. This led to a small sample size (n = 25). While many of the articles in the review (76%) were categorized by the researchers as Level 2, or quasi-experimental studies, each individual ambulatory care organization identified possessed its own inherent, unique challenges. As a result, the researchers were required to use broader-level thematic identification categories to best summarize the findings in the literature. Finally, a multitude of ACO models were included in this study (including two Medicaid ACOs). Future research on advanced ACOs, exclusive to model and risk-type is suggested.

## 6. Conclusions

The COVID-19 global pandemic has challenged the U.S. healthcare system and continues to stress provider organizations of all types. Ambulatory care organizations involved in ACOs with other healthcare organizations continue to support patient care during this challenging time, focusing on the safety and social distancing of patients and providers while CMS value-based reporting initiatives remain paused. This temporary relaxation of ACO reporting requirements allowed for an analysis of the literature to identify cost reducers and influencers attributed to ACO participation for outpatient organizations. While many outpatient organization characteristics may never return to pre-pandemic norms, this research utilized the quality reporting changes to identify what did work to control ACO-related costs, as well as what attributed to increased organizational costs.

Better (increased) coordination of patient care management initiatives, increased use of health information technology resources, and outpatient organization ownership models not associated with hospitals and/or ACO-participating hospitals are observations that have demonstrated a reduction of costs for outpatient organizations participating in ACOs. The presence and influence of social determinants of health and environmental conditions have been a contributing factor of increased costs to the outpatient organization in the ACO quality reimbursement environment. A lack of process and integration across ACO-participating organizations, as well as misaligned financial incentives for both providers and their organizations have also led to increased costs. As the U.S. works to control the spread of COVID-19 by way of vaccination and herd immunity, ambulatory care organization leaders are encouraged to reflect upon these ACO-related cost reduction and influencer practices as clinic practices begin their return to routine operations.

## Figures and Tables

**Table 1:** Summary of Findings (n = 25)**.**

Author(s)	Participant(s)	* JHNEBP Study Design	ACO Variables (Constructs) That Decrease Costs to Ambulatory Care Organizations	ACO Variables (Constructs) That Increase Costs to Ambulatory Care Organizations
Colla et al. [8]	Medicare (overall) population and clinically vulnerable sub-group.	2	No geographic variables (patient/ACO location) identified as a contributory variable in the study. Results are similar to the Physician Group Practice Demonstration and the Pioneer program across all patient groups (specifically < hospitalizations (ER) and <utilization overall).	Specific diagnoses (conditions) identified that significantly increased costs. Specific components and (care modality) significantly identified to increased costs. Vast differences in spending across ACO models identified. Greater structural changes recommended beyond utilization and hospitalizations required for enhanced patient outcomes.
D’Aunno et al. [9]	Medicare Shared-Savings ACOs	2	Establishment of relationship with local hospitals before ACO formation. Large (200+) provider groups with quality care established prior to ACO formation. Pre-established and meaningful EMR utilization already in-place. Use of care coordinators in the physician practice.	Distance between physician practices (geographic dispersion). Competition regarding primary care services between the physician practices and local hospital(s). Local hospital lack of awareness/identification of patients and the 30-day readmission criterium.
Fraze et al. [10]	Medicare Shared-Savings ACOs, focus on diabetes management	2	Organizations with multiple ACO contracts tended to perform better with diabetes management also. Organizations that offer more comprehensive services.	Integration with community health centers and/or hospitals. ACO performance on diabetes management decreased after contract year one (possibly due to transition from pay-for-reporting to pay-for-performance).
Gupta et al. [11]	Various UCLA clinic network patients with high expenditures: dementia, chronic kidney disease (CKD), and cancer	2	Leveraging of midlevel practitioners and care coordinators, health IT infrastructure, and other shared resources to reach the subpopulations of patients who may benefit most from specific interventions. Use of a patient health value (PHV) categorization to assist with identification of specific diagnosis needs and interventions.Use of a system wide PHV establishes a culture of value.	Fragmented care between UCLA network clinics. Initial lack of care goals documentation.
Hibbard et al. [12]	Primary care outpatient clinics with a high prediction of future utilization (ED visits and specific, future diagnoses).	3	Controlling baseline chronic disease status will prevent future utilization. Use of other opportunities to identify high-risk, high-utilization patients early. Incorporation of the patient’s ability for self-care/self-management. Investment in early interventions with high-risk patients does pay-off long term.	Focusing on clinic risk factors only, versus also incorporating system delivery challenges.
Ho et al. [13]	Percutaneous coronary intervention (PCI) patients in the VA health care system.	2	Outpatient care opportunities to explore differences in follow-up care, some of which may be related to the intensity of care provided, frequency of cardiac testing, and/or a need for noncardiac-related care.	Higher costs were associated with higher hospital utilization.
Hofler & Ortiz [14]	Rural health primary care clinics.	2	Consideration must be granted to demographic and limited clinical workforce make-up. Clear guidelines regarding primary care providers and related expectations in a rural environment.	Simply joining an ACO increases cost per visit, often up to or beyond two years. Incompatible EHR systems between organizations. ACO-related standardization costs for clinical personnel.
Horny et al. [15]	Clinics with patients enrolled in the diabetes specialty clinic with A1C ≥ 8.5% and at least one appointment no-show in the past year.	2	Use of non-clinical patient navigators to help improve both medical and administrative patient outcomes. Navigators keenly aware of specific patient needs and accommodating when scheduling appointments, limiting ED visits. Trained navigators as patient ‘peers’ versus healthcare providers.	Diabetes patients failing to schedule appointments, missing appointments, and had more unscheduled clinic visits (not part of patient navigation program). Alternative was ED utilization with compounded ailments.
Alhossan et al. [16]	Pharmacy for patients who recently received an annual wellness visit at a federally qualified health center participating in an ACO.	3	Utilization of clinical pharmacists during the annual wellness visit led to increased acceptance and utilization of recommendations for patients. Clinical pharmacist integration in the annual wellness visit allow for additional time freed-up for other medical providers. While additional screenings were recommended by the clinical pharmacist, this also led to additional revenue for the clinic.	Failure to integrate a clinical pharmacist in the treatment of patients in ACOs may forego additional benefits.
Koh et al. [17]	Longitudinal claims and enrollment data from the Massachusetts Medicaid pro-gram ACO.	2	Addressing patients’ medical, behavioral health, and case management needs in a home setting, versus in a clinic. Attention towards social determinants of health including homelessness needs to be built in both the ACO treatment protocols, and the financial reimbursement methods.	Homelessness identified as a significant variable (social determinant of health) for increased ACO spending. Frequent patient address changes and contact information.
Kralewski et al. [18]	2009 national survey of 211 group practices linked to Medicare claims data.	3	Physician owned and “other” owned practices are associated with better screening and quality measures than hospital owned practices. Better patient screening measures resulted in lower costs. Quality of care evaluation and ratings at the individual provider level.	Quality of care financial remuneration at the organization level only (group-level performance evaluation).
Lin et al. [19]	Medicare ACO claims data to analyze in and out of network specialty care.	2	Small changes to out of network primary care delivery can have large effects on overall organizational performance.	Increased levels of out of network specialty care for ACO patients ($10.79 increase in spending per beneficiary, per quarter). More out-of-network primary care was associated with higher total spending.
McConnell et al. [20]	Oregon and Colorado Medicaid ACO models.	3	A strong focus on manageable, incremental steps has been followed by growth in enrollment, reductions in utilization, and improvement in key performance indicators. Planning for future, additional efforts of additional utilization controls.	2014 Affordable Care Act Medicaid expansion efforts suspected to result in primary care capacity experienced. Short timeframe to transition to the ACO model leads to inefficiencies.
McWilliams et al. [21]	Medicare claims data of ACO programs.	2	Policy implication: In a one-sided contract without downside risk, an ACO that increases spending in one contract period is not penalized for doing so and is rewarded in the subsequent period with a higher benchmark. Questionable ‘gaming’ behavior suspected by healthcare organization’s selection of providers and/or patients that possibly led to increased ACO reimbursements.	Policy implication: An ACO that lowers spending in one contract period is disadvantaged in the subsequent contract period with a lower benchmark. Authors argue to disassociate the link between current benchmark performance and prior ACO savings.
McWilliams et al. [22]	Fee-for-service Medicare claims data to compare hospital-integrated ACOs versus physician group ACOs.	2	Physician group ACOs demonstrated significant reduction in savings.	Hospital-integrated ACOs showed no reduction in savings.
Navathe et al. [23]	n/a	3	Authors conclude “Extending the duration of the bundles, expanding the accountable entities beyond hospitals, and integrating bundled payments with global budget models within ACOs) better align episode-based payment with population health and offer a smoother path to budgets.”	Incongruent reimbursement models recognized for high historical baseline payments for patients that have poor care outcomes when integrated into an ACO bundle.
Bannon et al. [24]	University of Utah Health System Medicaid ACO high-risk patients.	2	Specific outpatient programs for care-intensive patients that are custom-tailored to the communities they serve. Re-direction of pre-identified, high utilization patients to an “intensive outpatient clinic” versus standard treatment localities.	While utilization of such high-utilization clinics has been conducted at other organizations, the authors cite a failure to further address patient outcomes and effective cost savings in the end.
Ouayogode et al. [25]	Survey information on care management and coordination processes linked to Medicare ACO claims data.	2	Suggested use of care navigators to assist with limiting readmissions and overall hospitalizations.	Failure to assess care coordination and management efforts.
Rosenthal et al. [26]	Medicaid claims/encounter data.	2	Utilize lessons learned from ACO pioneer programs and incorporate findings into local ACO program(s).	Under-resourced and highly regulated Medicaid models at the state level. Over-reliance upon claims data only, versus the incorporation of clinical-outcomes data.
Schumacher et al. [27]	Chronic heart failure patients at a large, networked medical group.	2	Expanded use of a clinical pharmacist to identify opportunities to better care for comorbidities. Use of a clinical pharmacist to develop heart failure and other treatment protocols to assist integrated providers. The clinical pharmacist was able increase the scope of practice and patient panels through physician referrals.	No cost increase variables.
Shah et al. [28]	Clinically integrated delivery system participating as a Pioneer ACO.	2	Use of increased telehealth resulted in a reduction of in-person patient visits, while increasing overall visits by 80%. A virtual visit program is able to limit overutilization, while also increasing access to care.	ACO provider organizations will bear the costs of new/updated telehealth implementation efforts (no health insurers, etc). The study notes caution to not assume a long-lasting reduction in in-person visits over a duration of time (eventually will plateau). Disparities surrounding patient demographics and access to telehealth technologies noted as a program disadvantage to some patients.
Beckman et al. [29]	Two primary care physician-led ACO organizations and Medicare beneficiaries receiving annual wellness visits.	2	Use of first-time annual wellness visits decreased overall organizational costs when compared to the control group. Patients receiving annual wellness visits to did seek care at a hospital had less severe illnesses than the control group. Incentivizing all stakeholders associated with primary care leads to cost reductions/savings.	Enhancing primary care services beyond “usual” care offers mixed results and not necessarily cost savings.
Blewett & Owen [30]	Hennepin (MN) Health Medicaid ACO	3	Enhanced use of patient care technology and data sharing led to a reduction in hospital visits and a slight (3.3%) increase in outpatient clinic visits. Requirement for better (ongoing) data sharing among state Medicaid organizations (payers) and health care organizations.	Lack of a state or national program for low income ACO populations cited as a concern for future risk.
Burgon et al. [31]	Comparison of regional ACO patient outcomes with non-participating organizations/patients.	2	Physicians in ACOs with evidence-based feedback significantly improved care and cost-efficiency. Improvements in the simulations correlated with im-proved performance in patient-level quality measures.	Lack of appropriate tools and provider feedback loops disallow the opportunity to improve during the quality reporting period(s).
Chang et al. [32]	Long-term Medicare nursing home patients attributed to an ACO model.	2	ACO long-term nursing home residents had less use of discretionary care. Continued opportunities to help reduce cost due to unnecessary utilization exists, as nursing home patients often generate a significant volume of E&M provider visits.	While fewer ED and other hospitalizations were identified for patients under the ACO attribute, cost reductions were not experienced. Patients switching providers frequently within an ACO reporting period can lead to cost ramifications beyond quality outcome reporting.

* Johns Hopkins Nursing Evidence-Based Practice (JHNEBP) levels of strength of evidence:
Level 1, experimental study/randomized control trial (RCT) Level 2, quasi-experimental study Level 3, non-experimental, qualitative, or meta-synthesis study Level 4, opinion of nationally recognized experts based on research evidence/consensus panels Level 5, opinions of industry experts not based on research evidence
